# Quality Improvement Intervention associated with Improved Lung Protective Ventilation Settings in an Emergency Department

**DOI:** 10.51894/001c.29603

**Published:** 2022-02-24

**Authors:** David H. Heimberg, Zachary Illg, William D. Corser

**Affiliations:** 1 Department of Emergency Medicine Henry Ford Wyandotte Hospital; 2 Department of Emergency Medicine Emory University School of Medicine; 3 Statewide Campus System Michigan State College of Osteopathic Medicine

**Keywords:** lung-protective ventilation, quality improvement, emergency medicine, ventilator management, mechanical ventilation, lung protective strategies

## Abstract

**INTRODUCTION:**

Patients requiring endotracheal intubation and mechanical ventilation in the emergency department (ED) are critically ill, and their ventilator management is crucial for their subsequent clinical outcomes. Lung-protective ventilation (LPV) setting strategies are key considerations for this care. The objectives of this 2019-2020 community-based quality improvement project were to: a) identify patients at greater risk of not receiving LPV, and b) evaluate the effectiveness of a series of brief quality improvement educational sessions to improve LPV setting protocol adherence rates.

**METHODS:**

A 15-month retrospective chart review of ventilator settings and subject characteristics (N = 200) was conducted before and after a series of 10-15-minute educational sessions were delivered to improve LPV adherence. This information was presented at a series of four educational sessions for 25 attending physicians (n = two sessions) and 27 residents at conferences (n = two sessions). Two additional materials (e.g., LPV reference charts, tape measures to gauge patients’ heights) were also posted in three ED resuscitation rooms and on cabinets containing emergency airway equipment. The pre and post-intervention occurrence rates of LPV setting orders were inferentially compared before and after educational sessions.

**RESULTS:**

Patients ventilated using LPV increased from 70% to 82% after the educational sessions (p = 0.04). All patients who were 67 inches or greater in height were ventilated appropriately before and after sessions. For patients under 65 inches in height, post-session LPV adherence increased from 13% to 53% (p = 0.01).

**CONCLUSIONS:**

Based on these results, a brief ED provider educational intervention can significantly improve the utilization of LPV guideline-based settings. Patients under 65 inches in height may also be especially at risk of receiving non-LPV ventilator setting orders.

## INTRODUCTION

Patients who present to the emergency department (ED) requiring endotracheal intubation and mechanical ventilation for respiratory failure or protection of their airway are critically ill, and their ED care is crucial for their subsequent clinical outcomes.^1^ Low tidal volume ventilation, also known as lung-protective ventilation (LPV), has been typically defined as less than or equal to 8 mL/kg of predicted body weight (PBW), as a strategy to improve outcomes in such patients.^2^

LPV has been associated with reducing mortality and pulmonary complications in patients with and without acute respiratory distress syndrome (ARDS).^1–4^ Although the harmful effects of high-tidal volume ventilation (i.e., > 10 mL/kg. PBW) have been widely accepted, the optimal tidal volume strategy for ED patients continues to be debated.^5^ Emergency departments that have implemented evidence-based LPV protocols have shown reductions in ventilator-associated complications (e.g., ARDS, ventilator-associated pneumonia, and mortality).^6^ Post-education improvements in adherence to LPV guidelines have also been shown in intensive care unit (ICU), ED, and operating room settings.^7–9^

### Purpose of Project

The objectives of this two-phase quality improvement project were to: a) assess the current implementation of LPV practices in the authors’ ED, and b) evaluate the effectiveness of a series of brief LPV protocol educational sessions for attending and resident physicians. To direct the delivery of the second-phase educational sessions, patient characteristics associated with not being treated with LPV were first identified. The overall null hypothesis of the study team was that they would be unable to measure any post-education improvements in LPV protocol adherence across patient sample subgroups.

## METHODS

### Setting and Study Design

This two-phase quality improvement project was conducted at an urban, community-based, 401‑bed hospital with an approximately 55,000 annual ED patient visits. In the ED, intubations were performed by emergency medicine residents or attending physicians with ventilator management overseen by physicians in coordination with respiratory therapists.

During the first project phase, retrospective electronic health record (EHR) data were extracted to evaluate the proportion of ventilated patients who had been treated using non-LPV ventilator settings in the ED. For the second phase, patient characteristics were also examined for possible sample subgroup (e.g., age group, gender) differences before and after completion of LPV educational sessions.

The full project window spanned approximately 15 months (i.e., December 6, 2018 to March 15, 2020) with 100 (50%) patient intubations occurring before the educational sessions. During the following four months, a series of four educational sessions was delivered, with the remaining five months of the study were dedicated to analysis of another 100 (50%) patients’ ventilation orders. Before data collection, the hospital’s institutional review board had approved the project design with expedited approval and waiver of informed consent.

### Study Population

An initial query of the physician authors’ EHR (EpicCare Link, Epic Systems Corporation, Verona, WI) was conducted identifying ED-ventilated patients who were 18 years of age or older and had been admitted to the intensive care unit (ICU). Exclusion criteria included mortality within 24 hours of ED intubation, enrollment in hospice from the ED, use of pressure-targeted ventilation, severe metabolic acidosis, and/or missing EHR ventilation setting data.

### Sample Size Calculations

Before the study, the authors had used G*Power 3.10.10 software^10^ to generate *a priori* minimal sample size calculations for the primary study endpoint: proportionate differences in pre-to-post session LPV adherence rates in the ED when stratifying patients into either pre or post-education subgroups. These calculations indicated that a minimum sample size of 164 patients would afford the study team with an adequate 0.80378 1 minus β level of statistical power to detect statistically significant pre- to post-education ventilator setting differences.

### Educational Intervention

A series of four educational sessions regarding LPV protocols were provided to 25 attending EM physicians (two sessions), and 27 resident EM physicians (two sessions) by the first and second authors. Study information and LPV reference materials were also later disseminated to respiratory therapists by the respiratory therapy manager. A single followup email reminder concerning guideline-based LPV practices was later disseminated to all sample providers.

Sessions lasted approximately 10-15 minutes and consisted of a brief review of the LPV research literature to date and a focus on the authors’ first-phase ED data results. The authors reported the greater proportion of shorter ED patients often being ventilated with higher non-LPV tidal volumes. Finally, the ready availability of disposable tape measures and LPV reference charts in the three ED resuscitation rooms and on cabinets containing emergency airway equipment were described.

The quick reference charts of PBWs and guideline tidal volumes by height/PBW at 6, 7, or 8 mL/kg were posted in the three ED resuscitation rooms, where nearly all intubations occur.^11^ Although participating providers (i.e., respiratory therapists, residents, and attending physicians), were each encouraged to adhere to suggested LPV settings, final ventilator settings were sometimes adjusted at the discretion of the attending physician.

### Outcomes of Interest

The primary outcome was the proportion of patients treated with LPV ventilator settings following intubation in the ED before and after the educational sessions. The physician authors chose a goal tidal volume of less than or equal to 8 mL/kg. based on PBW as consistent with the LPV research to date.^1,2,11^ Other patient characteristics (i.e., age in complete years, gender, body mass index [BMI], weight, and height) were also measured to evaluate their significance on documented LPV and non-LPV settings throughout the project.

### Data Analyses

All analytic procedures were conducted by the third author with over 98% complete data using *SPSS Version 25* (IBM, Armonk, New York).^12^ For continuous measures (e.g., age, height, BMI, and final ED ventilator settings), the analyst conservatively categorized continuous variables into equivalent-sized tertile subgroups. The overall distribution of the primary “final ED ventilator setting” study outcome had been first determined to be significantly non-parametric (i.e., non-normal) (Shapiro-Wilk statistic = 0.882, df 200, p ≤.001).

First, a series of simple chi-square and independent subgroup t-tests for independence were conducted for the selected dichotomous (i.e., “LPV ordered in ED” or “LPV not ordered in ED”) outcome of interest.^13^ Second, a series of Pearson r product-moment bivariate correlations stratifying patients by their personal characteristics (e.g., age, gender, etc.), including pre and post-presentation status as a prospective predictive modeling term in the final regression models.^14^

Finally, the analyst completed an additional series of controlled non-parametric binary logistic regression “stepwise” (i.e., each model term introduced individually, retained in initial p value < .10) multivariate predictive models to examine the relative significance of pre- or post-education status controlling for patient characteristics on LPV ventilator setting differences.^15^

## RESULTS

**Figure 1.**
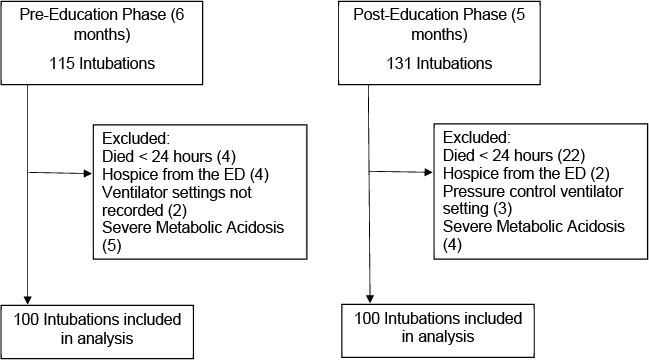
Flow Diagram

Data from a total of N = 246 patients who had been intubated in the ED were first considered for inclusion in the analytic sample (Figure 1). A total of 46 (18.7%) patients were excluded from the sample based on pre-determined exclusion criteria as follows: 26 (56.5% of excluded) experienced mortality within 24 hours of presentation, six (13.0%) were enrolled in hospice while in the ED, nine patients (19.5%) experienced severe metabolic acidosis, three (6.5%) were ventilated using pressure-control ventilation, and two patients (4.3%) were excluded due to missing ventilator data. Data from the remaining 200 (81.3% of initially identified) patients (i.e., 100 pre-education and 100 post-education) were therefore included in data analyses.

No statistically significant differences were demonstrated between the pre- and post-educational project subgroups (Table 1).

**Table 1.** Patient Characteristics by Project Phase

	**Pre-Education Phase** **(N = 100)**	**Post-Education Phase** **(N = 100)**	**P value**
**Age, mean (SD), years**	61.93 (SD = 15.49)	60.30 (SD = 16.84)	0.23
**Male sex, %**	46%	43%	0.70
**Height, mean (SD), inches**	66.7 (SD = 4.68)	66.3 (SD = 4.10)	0.50
**BMI, mean (SD), kg/m^2^**	30.2 (SD = 9.58)	28.37 (SD = 10.01)	0.55
**PBW, mean (SD), kg**	62.77 (SD = 12.45)	61.93 (SD = 11.10)	0.57
**Initial Lactate**	4.72 (SD = 4.57)	4.61 (SD = 4.82)	0.32

### Patient Characteristics

The mean age of patients (N = 200) at time of ED intubation was 66.11 years (SD 16.16), with ages ranging from 18 to 98 years. A total of 111 (55.5%) patients were female. The average BMI of sample patients was 29.38 kg/m^2^ (SD 9.82), ranging from 13.31 to 68.30. A total of 152 (76.0%) sample patients from both project phases were ventilated using LPV settings.

### LPV Utilization

**Figure 2.**
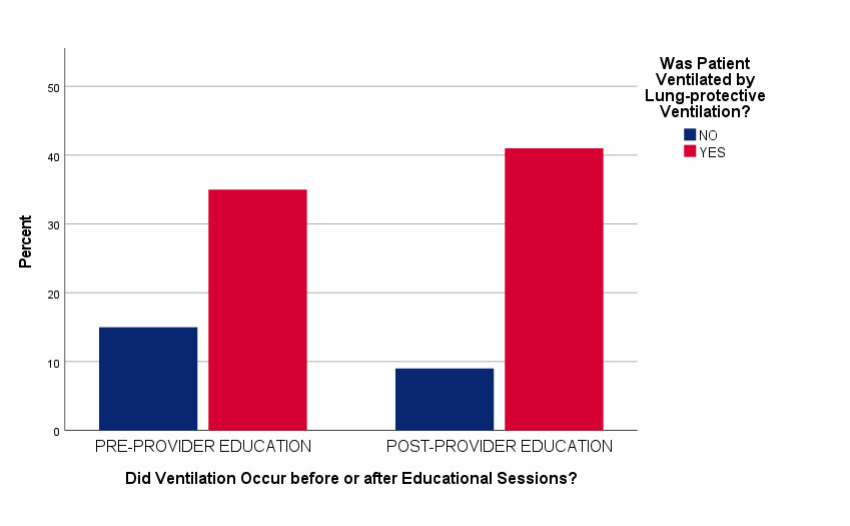
Proportions of Lung-Protective Ventilation by Project Phase (N = 200)

During the pre-intervention project phase, 70 (70%) patients were ventilated using LPV settings (≤8 mL/kg PBW). After project intervention, 82 (82%) patients were ventilated using LPV, representing a statistically significant overall increase in LPV utilization without controlling for other patient characteristics (Pearson X^2^ = 3.947, df 1, p = 0.04) (Figure 2).

### Multivariate Predictive Models for Tidal Volumes (total sample)

When controlling for each measured patient characteristics (e.g., age group, gender, BMI) in predictive models, the earlier-significant differences in mean pre and post-educational tidal volumes fell out of statistical significance (Wald = 2.747, df 1, p = 0.10). Mean pre-education tidal volume was 7.50 mL/kg (SD = 1.22), and mean post-education tidal volume was 7.25 mL/kg of (SD = 1.06). This finding indicates that patient characteristics likely accounted for a larger proportion of pre-post-education ventilator setting differences than the educational sessions did across the sample.

For example, BMI category was a significantly predictive variable associated with whether an ED patient was ventilated utilizing LPV (Wald = 7.258, df 1, p = .027) (Other model terms: Tertile age category p = 0.76, gender p = 0.29, and ED respiratory rate category p = 0.91).

### Subgroup Analytics (Patient Heights Less than 65 Inches Only)

**Figure 3.**
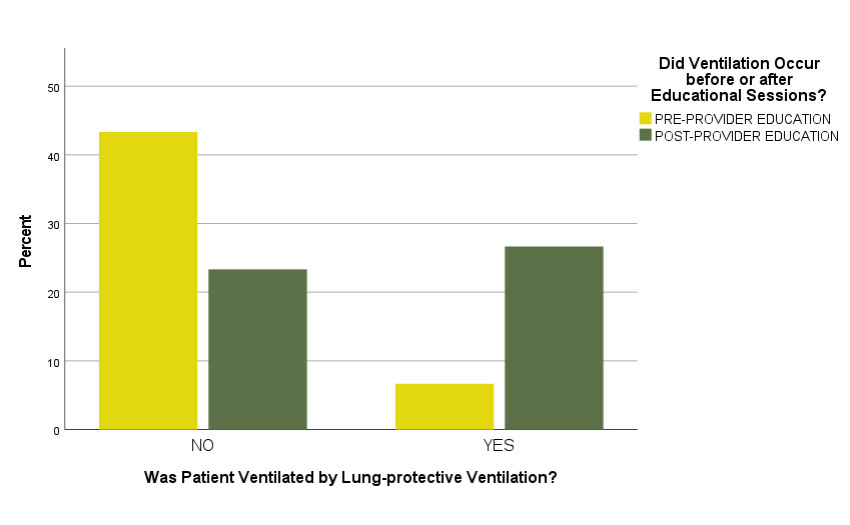
Proportions of Lung-Protective Ventilation by Project Phase in Patients with Height <65 Inches

The physician authors initially noted that all ED patients above 67 inches in height were ventilated according to LPV standards both before and after the educational sessions were delivered. Additional subgroup analyses of those subjects in the lowest tertile by height (< 65 inches) were therefore also later conducted. In the 60 (30%) of total sample patients with heights < 65 inches, four of 30 (13.3%) were ventilated utilizing LPV prior to the educational sessions, and 16 of 30 (53.3%) were ventilated utilizing LPV following the educational sessions (Figure 3). In logistic regression models, this represented a statistically significant increase in adherence to LPV guidelines when controlling for patient characteristics (Wald = 8.046, df 1, p = 0.01).

## DISCUSSION

Based on these project findings, educating ED providers concerning LPV ventilator setting guidelines may be a relatively simple intervention to improve clinical outcomes for ventilated patients. Prior studies in academic medical centers have demonstrated similar improvements in ventilator setting adherence,^9^ as well as improvements in patient mortality and ventilator-associated complications.^6^ As community hospitals may not have overnight on-site intensivists to adjust initial ED settings, the implementation of evidence-based ventilator management practices in the ED may be especially crucial.^8^

As previously reported, all sample patients above 65 inches in height were ventilated utilizing LPV both before and after the educational sessions. Our educational intervention was associated with a statistically significant increase (p = 0.01) in the proportion of patients under 65 inches who were ventilated using a lung-protective strategy.

In 2021, higher adherence to LPV than typically seen in previous studies was found by Foley et al. both before and after their implementation of a mechanical ventilation protocol.^15^ The results of this and our 2019-2020 project may reflect an overall trend among emergency physician or respiratory therapists towards greater adoption of using low tidal volumes. Adopting an EHR-based ordering set that incorporates a patient’s height has also been shown to help overcome non-LPV provider practice patterns.^16^

More robust protocols to measure patients’ heights and calculating their tidal volumes should also be considered for future studies.^7–9^

We experienced several logistical difficulties when delivering project presentations. Many attendings, residents, and respiratory therapists worked a combination of morning, evening, and overnight shifts which posed an obstacle when attempting to deliver scheduled group sessions. After the project, we also concluded that assimilating such practice changes were also more difficult for staff (e.g., respiratory therapists) who were requested to incorporate such practice changes across multiple hospital units.

### Study Limitations

Our use of retrospective EHR data from a convenience sample at a single community-based hospital may limit the generalizability of our results to other EDs. Our study focused on tidal volume ventilation, not other aspects of LPV and best ventilator care (e.g., use of plateau pressures, PEEP / FiO_2_ tables, etc.) or subsequent post-ED patient outcomes (e.g., mortality, ARDS, ventilator associated pneumonia rates). Our pre-post project results may have been prone to unmeasured biases, and the long-term sustainability of presentation results were not tested.

## CONCLUSIONS

Our project findings suggest that a series of relatively brief educational sessions with provision of appropriate LPV ordering tools can serve to improve ventilator management of intubated patients, particularly for patients of shorter height. Future larger-sample studies are needed to identify the numerous factors influencing ventilator ordering processes in the ED. The lessons derived from this quality improvement project may prove useful to other institutions looking to improve care for critical ventilated patients at highest risk.

### Conflicts of Interest

None

### Financial Support

None
